# Elastic Modulus Distribution in Rigid PU Foams of Elliptically Graded Density

**DOI:** 10.3390/polym18202549

**Published:** 2026-10-16

**Authors:** Ilze Beverte, Ugis Cabulis

**Affiliations:** Latvian State Institute of Wood Chemistry, Dzērbenes Street 27, LV-1006 Riga, Latvia; a09660418@gmail.com

**Keywords:** polyurethane foams, graded density, elastic modulus, distribution map, ellipse, aspect ratio

## Abstract

Structural and insulating components for automobiles, marine vessels, aircraft, spacecraft and other vehicles and technical devices are often manufactured by foaming rigid polyurethane foams in sealed molds; consequently, it is crucial to understand the physical and mechanical properties of the molded foams. The objective of the study is to evaluate the elastic modulus distribution in rigid polyurethane foams with an elliptically graded density that appears when foaming in a parallelepiped-shaped sealed mold at overpressure. The dependence of the modulus on the density was investigated experimentally under compression, and the constants of the model function were determined. Using the constants, the modulus values were calculated and corresponding distribution maps were plotted for vertical and horizontal cross-sections of the foam block. The results of numerical calculations showed that the modulus distribution is elliptical in vertical sections of the block and circular in horizontal cross-sections. It was concluded that the elliptical symmetry of the density distribution leads to symmetry of the same kind in the elastic properties. The modulus distribution maps are model-based predictions which require an independent experimental spatial validation. The dependence of the aspect ratio of the equidensity and equimodulus ellipses on the aspect ratio of the mold was estimated.

## 1. Introduction

Rigid polyurethane (PU) foams are polymer–gas composites with excellent thermal insulation properties and acceptable load bearing capacity, which are used in various engineering solutions [1,2,3,4,5]. The foams are applied in the construction sector in sandwich structures and panels, in the transport for cooling vans and vehicle insulation, as cryogenic insulation in aircraft and spacecraft, for both heat- and cold-saving insulation in stores, warehouse buildings, and constructions, in wind rotor blades and steering rudders of sailing yachts, etc. [6,7,8].

Rigid PU is a thermosetting polymer network synthesized using highly branched, short-chain polyols and polymeric isocyanates, which contain several specific chemical linkages: primary covalent bonds (the backbone and crosslinks) and secondary intermolecular interactions (the physical network) [1,2]. The rigid PU network ensures the mechanical stiffness, high thermal stability, and structural integrity of PU foams. Recent advances in manufacturing technologies have led to the development of cellular solids with controllable microstructures and properties modulated to perform multiple functions at low structural weights [9,10]. The plastic foam properties are controlled by creating a density gradient in the foam structure to locate the material where it is mostly needed, while keeping the total weight constant [11]. A 3D-printing procedure to fabricate tailored honeycomb structures from thermoplastic polyurethanes (TPU) was developed in [11,12]. By introducing the density grading, the energy absorbing and damping profiles were significantly modified from the uniform density equivalent. Three-layered graded structures fabricated from rigid PU foams with nominal densities of 160 kg/m^3^, 240 kg/m^3^, and 320 kg/m^3^ were studied at quasi-static and dynamic compressive loadings in [13] using the digital image correlation to characterize deformation and strain fields developed within the layers.

The study [14] investigated how the density differences between core layers and their specific arrangement affected the blast response of graded PU foam cores under the simultaneous constraints of equivalent total mass and overall thickness. Ungraded and graded foam core panels were analyzed and compared based on deformation and energy absorption. In [15], application of functionally graded (FG) foam materials inside the rotor blades or wings of an unmanned aerial vehicle to vary their stiffness was studied. A simplified full-scale model of a helicopter blade with the distributed gradient properties of foam material inside the structure was studied numerically.

Density-graded PU foams are often made in sealed molds in industrial production and labs [1,2,3], e.g., in [16,17,18], sealed molds of two basic shapes—rectangular and cylindrical—were used in manufacturing rigid PU foams in lab conditions, but the grading of density and elastic modulus was not estimated prior to mechanical testing. In [19], microscopy of a structure showed that uniform local spatial distribution of the polymeric struts existed alongside graded density. The density distribution was estimated within a rigid PU foam block manufactured in a rectangular sealed mold, at overpressure. Corresponding maps with elliptical density zones and equidensity lines of the locally isotropic foams were plotted, but elastic modulus was not yet considered. In manufacturing of sandwich panels, a sealed mold and overpressure were used to get an isotropic PU foam core [20]. It was concluded that closeness of the foams’ structure to the isotropic one depends on how high the overpressure is. In sandwich panels manufactured at a lower overpressure, an anisotropic structure was identified.

Sealed molds are particularly useful in studies of PU foams filled with nanoparticles like spherical TiO_2_, platelet–nanoclays, rod-shaped carbon nanofibers, etc. [21,22]. When neat and nanoparticle-filled PU foams are produced in an open mold [23,24], the degree of monotropy of the filled PU foams differs from that of neat foams, because the foams rise to different heights [25]. The volume of foam blocks manufactured in a sealed mold is constant for all compositions and the locally isotropic structure permits comparing properties [26,27,28,29,30].

The objective of the study is to develop a method for assessing the elastic modulus distribution within a rigid PU foam block produced via confined foaming in a rectangular, sealed mold, at overpressure. The core concept involves correlating density distribution maps with the empirical relationship “density–modulus”. This approach eliminates the need for direct testing on every volumetric element of the PU foams’ block, a process that would otherwise entail significant expenditures of time, energy, and raw materials. The dependence of the modulus on the density of the PU foam composition was investigated experimentally in compression, parameters of the model function were determined and the elastic modulus distribution maps were plotted. It was found that the modulus distribution is elliptical in the vertical central cross-section of the molded block and circular in the horizontal cross-sections. A relationship between the aspect ratio of the mold and the equidensity and equimodulus ellipses was estimated for the given foaming conditions. A parametric equation of equimodulus lines in spherical coordinates was derived for any point of the vertical section.

## 2. Materials and Methods

### 2.1. PU Foams’ Blocks

A block of conventional closed-cell rigid PU foams was produced by confined foaming in a sealed plywood mold, using the chemical composition given in Table 1 and the technology [19]; dimensions of the block with skins were L_01_ × L_02_ × L_03_ = 250 mm × 250 mm × 130 mm.

The produced block was sawn in half by a vertical, centered cut, and the cut surfaces were assessed visually. A skin of significantly denser foams than the core foams was detected, and its thickness ΔL was measured using ISO 845:2006 [31]. After trimming the skin with a thickness of ΔL ≈ 10 mm … 15 mm from the entire outer surface of the block, a parallelepiped with dimensions of L_1_ × L_2_ × L_3_ = 230 mm × 230 mm × 100 mm and an apparent core density of 96.2 kg/m^3^ was obtained, where L_i_ = L_0i_ − 2ΔL and i = 1, 2, 3.

To provide a sufficient number of experimental data points for approximating the relationship between the elastic modulus and density of PU foams at densities <100 kg/m^3^, another block of foams was produced using the same chemical composition and technology, but the amount of physical blowing agent (pentane) was increased to 35 pbw. After trimming the skins, a parallelepiped with dimensions of 230 mm × 230 mm × 100 mm and an apparent core density of 56.4 kg/m^3^ was obtained.

### 2.2. Compression Test

**Figure 1:**
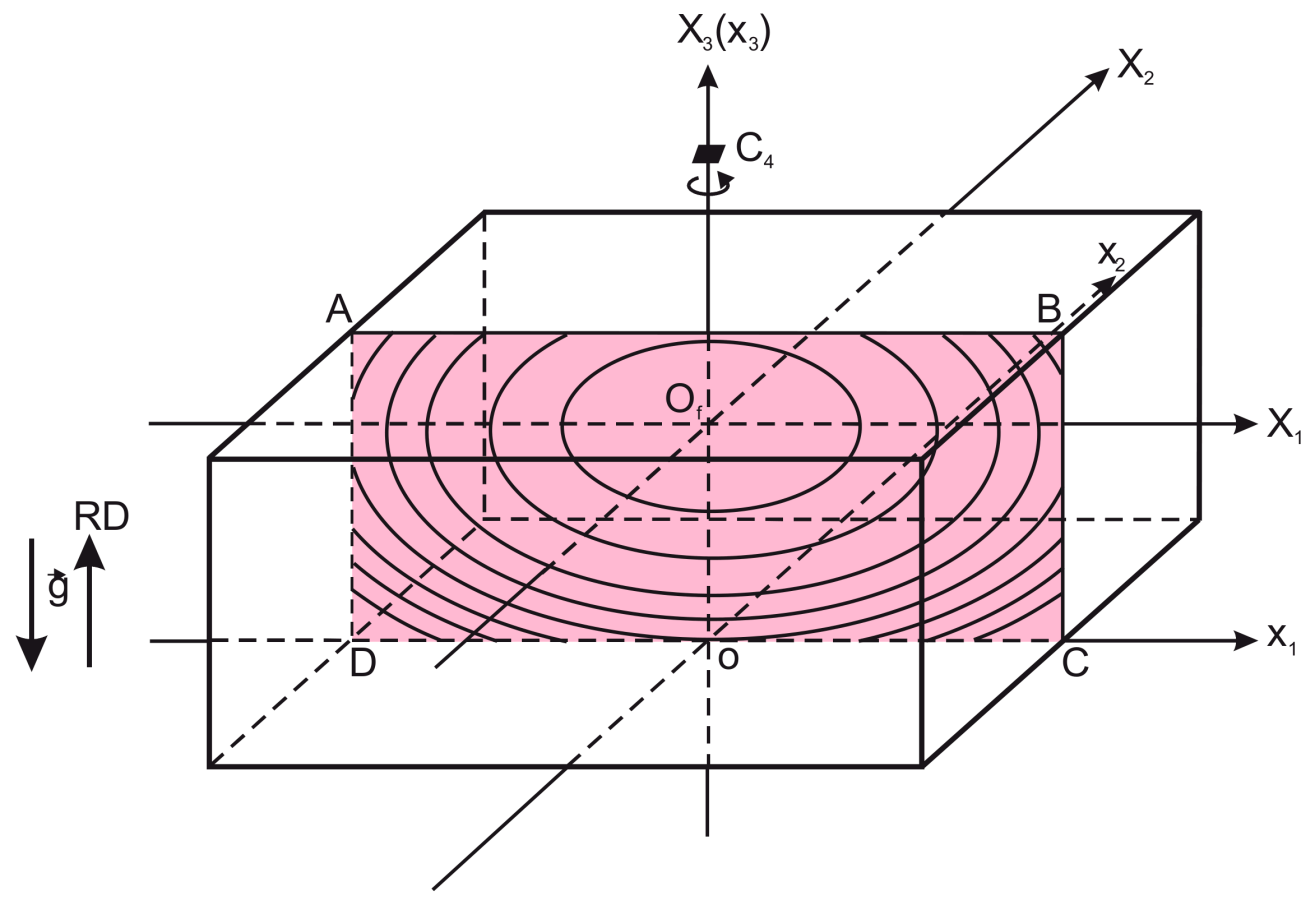
The PU foams’ block and equimodulus lines in the X_1_O_f_X_3_ section plane (pink); **g** is the Earth’s gravity vector.

Two Cartesian coordinate systems were used: x_1_ox_2_x_3_ and X_1_O_f_X_2_X_3_ (Figure 1). The x_1_ox_2_ plane of the x_1_ox_2_x_3_ system coincides with the bottom face of the trimmed block. It was established in [19] that the density distribution within a rigid PU foam block (produced in a rectangular sealed mold, at overpressure) exhibits elliptical symmetry with respect to point O_f_ (the foaming center), which is located app. 17 mm above the geometric center O_gc_ of the block. It is expedient to analyze any type of symmetry (cylindrical, spherical, etc.) relative to its characteristic axes; therefore, an additional coordinate system X_1_O_f_X_2_X_3_ was introduced, with its origin at point O_f_ and axes oriented along the semi-axes of the equidensity ellipsoids, as shown in Figure 1.

The elastic modulus and the density of isotropic PU foams are linked by an empirical relationship:

E = Aρ^d^, (1)

where A and 1 ≤ d ≤ 2 are constants that depend primarily on foams composition [3,4]. To determine “A” and “d” for the given composition, a compression test was conducted.

It has been demonstrated microscopically in [19,22] that the cellular structure of the rigid PU foams produced in a sealed mold, at overpressure, is locally isotropic. It implies that, given sufficiently small dimensions of the partition elements, the elastic modulus of an element or a group of elements is independent of the loading direction. The location of test samples within the two produced blocks (with apparent core densities of 56.4 kg/m^3^ and 96.2 kg/m^3^) and the measuring methodology were selected to ensure measuring of the displacement on a base of isotropic material.

It can be seen in Figures 5 and 6 of [19] that a 3D region of nearly constant density and modulus exists within the block around its foaming center O_f_. The test samples (rectangular prisms measuring 30 mm × 30 mm × 60 mm) were extracted from this region. The samples were oriented such that their height h = 60 mm was symmetrical with respect to the plane X_1_O_f_X_2_ and parallel to the rise direction RD of the foams, as shown in Figure 1. The samples were located in three adjacent rows, three samples per row. The rows were located parallel to the X_2_O_f_X_3_ plane, on one side of it. The three samples, extracted from each of the three rows of a block, form a statistical selection from the corresponding population; altogether, six populations were considered (three in each block).

The length of measurement bases (the gauge lengths) was chosen to be sufficiently small to ensure determination of the displacements Δx_1_ and Δx_3_ on a nearly isotropic material: (a) 30 mm in the axial direction, and (b) 20 mm in the transverse direction. With the size of partition elements being 5.6 mm × 5.6 mm, the 30 mm measurement base length spans over app. 6 elements, while the 20 mm base of length spans over app. 4 elements. Analysis of the modulus distribution maps, as shown in Figures 5 and 6 of [19], leads to the conclusion that, over these distances, the modulus varies insignificantly both in the X_1_O_f_X_2_ plane and along the O_f_X_3_ axis.

Stress–strain curves were recorded in the axial and transverse directions on the Z100 TEW testing machine (Zwick GmbH & Co. KG, Ulm, Germany) at a strain rate $\dot{\varepsilon }=\;$5%/min–6%/min and an ambient temperature T = 23.5 °C. Displacements Δx_1_ and Δx_3_ were measured using a videoXtens 2-120 HP video extensometer (ZwickRoell, Ulm, Germany) and a uEye 01-3483CP-M-GL camera (IDS Imaging Development Systems GmbH, Obersulm, Germany), focused on the corresponding bases, located in the middle third of the sample height. Three samples were tested for each data point.

Prior to the compression test, the axial measurement base was marked on each sample and its linear dimensions were measured in accordance with EN ISO 1923 [31]. When the stress–strain curves entered the non-linear region, the tests were terminated. The part of the sample which corresponded to the axial measurement base was extracted from the sample, its mass was determined and the density was calculated using the initial linear dimensions.

The elastic modulus E = E_3_ was determined from the initial linear portion of the stress–strain curves according to ISO 844:2021(E) [32]. The experimental data were approximated by MS EXCEL v16.0 software (Microsoft Corporation, Redmond, WA, USA) Trendline tool, power function E = Aρ^d^. Poisson’s ratio was calculated as ν_31_ = −ε_11_/ε_33_, where ε_33_ and ε_11_ are the axial and transverse strains.

### 2.3. Statistical Analysis

Based on prior experience, it was assumed that the probability distribution for the random variable, the elastic modulus, was normal for each of the six populations. Each estimate of the modulus was based on n = 3 statistically independent observations; the number of degrees of freedom ν = n − 1 = 2. The statistical selections were small (n = 3 < 30); therefore, the t-coefficient from Student’s distribution was used to calculate the upper and lower confidence limits for the modulus estimates. The t-coefficient for a 95% confidence level was determined from [33,34], based on the t-distribution evaluated for a coverage probability of 95.45%, at ν = 2: t = 4.303. Since the standard deviations s were known only for the selections, the corresponding margin of error was calculated as MOE_γ_ = t $\frac{s}{\sqrt{n}}$, and the bounds of the confidence interval for the population mean as CI = $\bar{E}\pm {\mathrm{MOE}}_{\gamma }$, where γ is the confidence level.

Confidence intervals for the parameters of the trendline E = Aρ^d^ were calculated for the 95% confidence level [35,36]. The power function was linearized by taking the natural logarithm of both sides of the equation lnE = lnA + dlnρ. This transforms the non-linear power model into a standard linear regression model, where ln(A) acts as the intercept and d as the slope. The confidence intervals were calculated for the log-transformed parameters by the MS EXCEL v16.0 software (Microsoft Corporation, Redmond, WA, USA) Analysis ToolPak add-in, the Data/Data Analysis/Regression tool. The calculated bounds were exponentiated to convert them back into the original scale, to the confidence intervals of the trendline parameters A and d.

### 2.4. Maps of Elastic Modulus Distribution

With known constants A and d in Equation (1), the elastic modulus was calculated for the density maps from [19]. Due to the fourth-order rotational symmetry C_4_ of the foam block about the O_f_X_3_ (or ox_3_) axis [19,26,28,37,38], as shown in Figure 1, the modulus was calculated only for the first quadrants of the (1) horizontal layers, which are parallel to the X_1_O_f_X_2_ (or x_1_ox_2_) plane (size of elements ΔX_1_ × ΔX_2_ = 5.6 mm × 5.6 mm and matrix size [21 × 21] elements) and of the (2) vertical section X_1_O_f_X_3_ (or x_1_ox_3_) plane (size of elements ΔX_1_ × ΔX_3_ = 4 mm × 4 mm and matrix size [30 × 26] elements), as shown in Figure 1.

The obtained range of modulus values 24 MPa < E < 40 MPa was divided into n = 8 zones of width ΔE = 2 MPa, as shown in Table 2. The lower boundary of a zone belongs to that zone, and the upper boundary belongs to the next zone: E_n_^L^ ≤ E < E_n_^U^, n = 1, 2, …, 8. To plot the maps of the modulus distribution, elements in each zone were assigned a certain shade of gray; Table 2. The zones were numbered from the foaming center O_f_ to the periphery of the section plane.

### 2.5. Plotting the Equimodulus Lines

**Figure 2:**
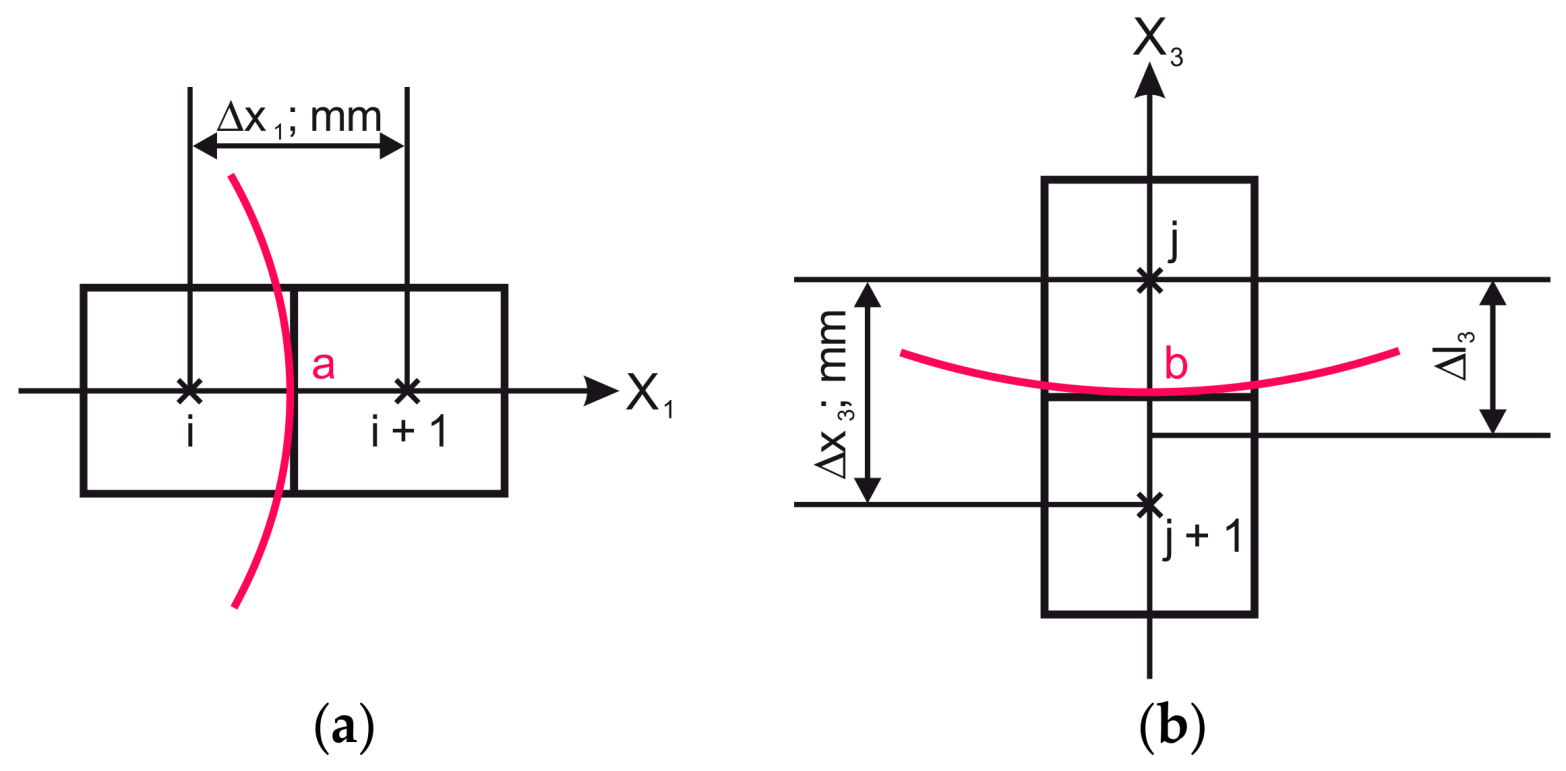
Determining of the (**a**) semi-major axis a and (**b**) semi-minor axis b of an equimodulus ellipse (red curves).

In the functional dependency between modulus and density, Equation (1), each modulus value corresponds to exactly one density value, so the equimodulus line corresponding to the equidensity ellipse is also an ellipse. To plot the equimodulus lines for the eight zones, two assumptions were made: (1) the modulus value of a partition element is assigned to its geometric center, as shown in Figure 2a, and (2) the modulus at a point on the coordinate axis, which connects the geometric centers of two adjacent elements j and j +1 (corresponding to modulus E_j_ and E_j+1_), depends linearly on the distance Δl_3_ of the point from element j, as shown in Figure 2b.

First, the semi-major axis “a” was marked on the O_f_X_1_ axis, at the border between two adjacent elements i and i + 1 from two adjacent density zones, as shown in Figure 2a. The modulus at the border was calculated as follows:

(2) $$E\left(X_{1}=a\right)=E_{a}=\frac{1}{2}\left(E_{i}+E_{i+1}\right).$$

The corresponding coordinate of the semi-major axis “a” equals $X_{1a}=X_{i}+\frac{1}{2}∆X_{1}$, where ΔX_1_ is the size of an element along the O_f_X_1_ axis. Now, the X_3_ coordinate of the semi-minor axis “b” should be marked on the O_f_X_3_ axis, between the centers of two adjacent elements j and j + 1 from two adjacent zones, as shown in Figure 2b. The modulus at the point X_3_ = b was calculated as

(3) $$E\left(X_{3}=b\right)=E_{b}=E_{j}+\frac{\left(E_{j+1}-E_{j}\right)}{∆X_{3}}∆l_{3},$$

where ΔX_3_ = ΔX_1_ is the size of an element along the O_f_X_3_ axis. From the definition of an equimodulus line as E(X_1_, X_3_) = const. for all X_1_ and X_3_, it follows E_b_ = E_a_. Taking into account Equation (2), the distance Δl_3_ can be expressed from Equation (3):

(4) $$∆l_{3}=\frac{E_{a}-E_{j}}{E_{j+1}-E_{j}}∆X_{3}.$$

Then, the coordinate of the semi-minor axis b on the O_f_X_3_ axis equals X_3b_ = X_j_ + Δl_3_. Knowing the coordinates of the vertex “a” and co-vertex “b”, an elliptical equimodulus line was plotted using the equation of an ellipse $\frac{X_{1}^{2}}{a^{2}}+\frac{X_{3}^{2}}{b^{2}}=1.0$, as shown in Figure 1. A similar methodology was used for plotting of equimodulus lines in the six horizontal layers.

### 2.6. Aspect Ratios of the Equidensity and Equimodulus Ellipses

Let us evaluate the dependence of the aspect ratio χ = b/a of (1) equidensity ellipses and (2) equimodulus ellipses on the aspect ratio of the mold χ_m_, where “a” and “b” are the semi-major and semi-minor axis of an ellipse. The aspect ratio of the mold (the working volume) is defined as χ_m_ = L_03_/L_01_ = L_03_/L_02_ = 130 mm/250 mm = 0.52 for all values 0.0 ≤ ξ_1_ ≤ 1.0 of the dimensionless coordinate ξ_1_ = X_1_/(L_01_/2). Assuming that the shrinkage of the foams is insignificant, the aspect ratio of the untrimmed block of foams is equal to the aspect ratio of the mold: χ_bu_ = χ_m_ = 0.52.

The density distribution map in the vertical section X_1_O_f_X_3_ of the trimmed block, given in [19] at size of elements ΔX_1_ × ΔX_3_ = 5.6 mm × 5.8 mm, was considered to determine the aspect ratio χ_d_ of equidensity ellipses. Numerical calculations showed that this size of elements is not small enough to determine the aspect ratio χ_d_ and to clearly outline the dependence of χ_d_ on the coordinate ξ_1_. A refined density map in the X_1_O_f_X_3_ section was plotted for the size of elements ΔX_1_ × ΔX_3_ = 4 mm × 4 mm (Figure S1 in Supplementary Materials), based on the methodology given in [19]. The equidensity lines were plotted, and semi-axes “a” and “b” were determined for ellipses № 1–9, for which both semi-axes were visible on the map. The aspect ratio χ, linear eccentricity c, and eccentricity e of the e ellipses were calculated: χ = b/a, c = $\sqrt{a^{2}-b^{2}}$, and e = c/a = $\sqrt{1-\frac{b^{2}}{a^{2}}}$. Semi-axes “a” and “b” were then determined for those equimodulus ellipses for which both “a” and “b” were visible in the modulus distribution maps in the vertical section X_1_O_f_X_3_ plane, at size of elements ΔX_1_ × ΔX_3_ = 5.6 mm × 5.8 mm.

The refined density map, size of elements ΔX_1_ × ΔX_3_ = 4 mm × 4 mm, was used to plot the equimodulus map in the vertical section X_1_O_f_X_3_ plane.

## 3. Results and Discussion

### 3.1. Results of Compression Test

The results of PU foams’ compression tests are presented in Table 3 along with the standard deviation (±s) and coefficient of variations (v; in %) of the statistical selections.

The values of Poisson’s ratio indicate a locally isotropic material in each row of samples of the two blocks; ν ≈ ν_31_. It confirms the results of the structural microscopy of rigid PU foams produced in a sealed mold [19,22]: a uniform local spatial distribution of the polymeric struts exists alongside graded density. These observations are consistent with the assumption of local isotropy under the investigated conditions.

**Figure 3:**
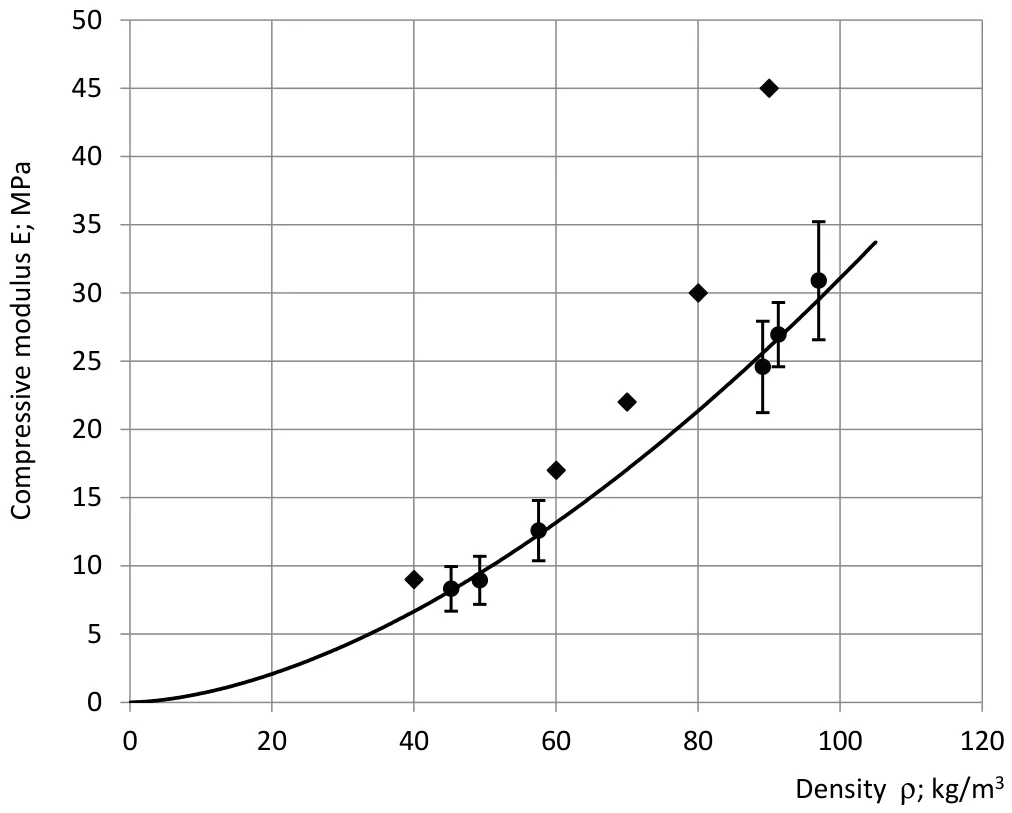
Experimental data of the elastic modulus in compression of PU foams (•) and the model function (the continuous curve); the bars show confidence intervals, and (♦) refers to experimental data from [3].

The mean values of density and moduli for the six selections are given in Figure 3 together with the confidence intervals for the mean values of the corresponding moduli populations. The confidence interval is the range of modulus values that likely contains the true population value; this means that 95% of repeated intervals will capture the true (but unknown) modulus value. To narrow the confidence intervals, larger selections of samples should be tested.

The dependence of the compressive modulus on density was approximated by a power function E = Aρ^d^ with parameters A = 0.0135 and d = 1.680, as shown in Figure 3. The correlation coefficient for the trendline R^2^ = 0.998. The calculated confidence intervals of the parameters A and d are 0.0127 ≤ A ≤ 0.0143 and 1.6668 ≤ d ≤ 1.6952. If we were to repeat the selections 100 times, the true population parameters would fall within the calculated ranges 95 times.

For comparison, data for conventional rigid PU foams from [3] are presented.

If samples of locally isotropic, density-graded PU foams contain elements that differ significantly in density and modulus (high density and modulus gradients), then the experimentally determined modulus values may depend on the length of measurement base, the configuration of density zones, and the loading direction.

### 3.2. The Calculated Maps of Elastic Modulus Distribution

**Figure 4:**
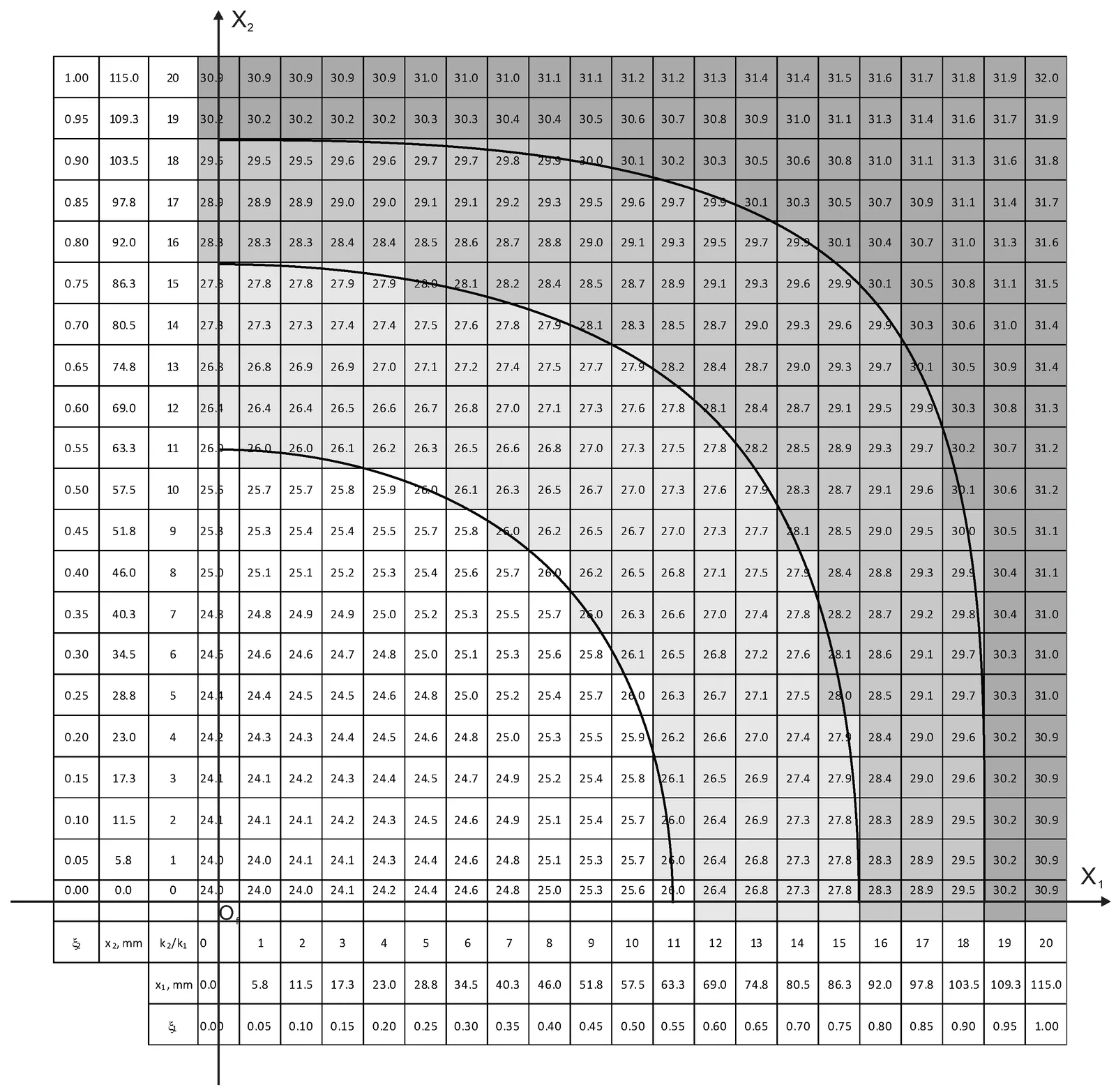
The calculated distribution of elastic modulus in horizontal layer № 4 (X_1_O_f_X_2_ plane; size of elements ΔX_1_ × ΔX_2_ = 5.6 mm × 5.6 mm).

The calculated maps of the elastic modulus distribution in horizontal layers № 1–6 are given in Figures S2–S4 of the Supplementary Materials. Map of the layer № 4, which includes the foaming center O_f_, is shown in Figure 4 as an example.

It can be seen that the central equimodulus zone of layer № 4 is circular, with the center at the origin of X_1_O_f_X_2_ coordinate system. Toward the sides of the block, the outer shape of the zones tends toward a square with rounded corners due to the influence of the rectangular shape of the mold. The modulus values are lowest in the central zone and highest in the corners for all layers. Layer № 1 has three equimodulus zones, while layers № 2–6 have four. The lowest modulus was identified in layer № 4 (24.0 MPa; in the central zone) and the highest in layer № 1 (35.7 MPa; in the corners). Distribution of the modulus is similar in layers № 4 and № 5; however, the foaming center O_f_ is located in layer № 4, as its central circular zone has a bigger radius than that of layer № 5.

**Figure 5:**
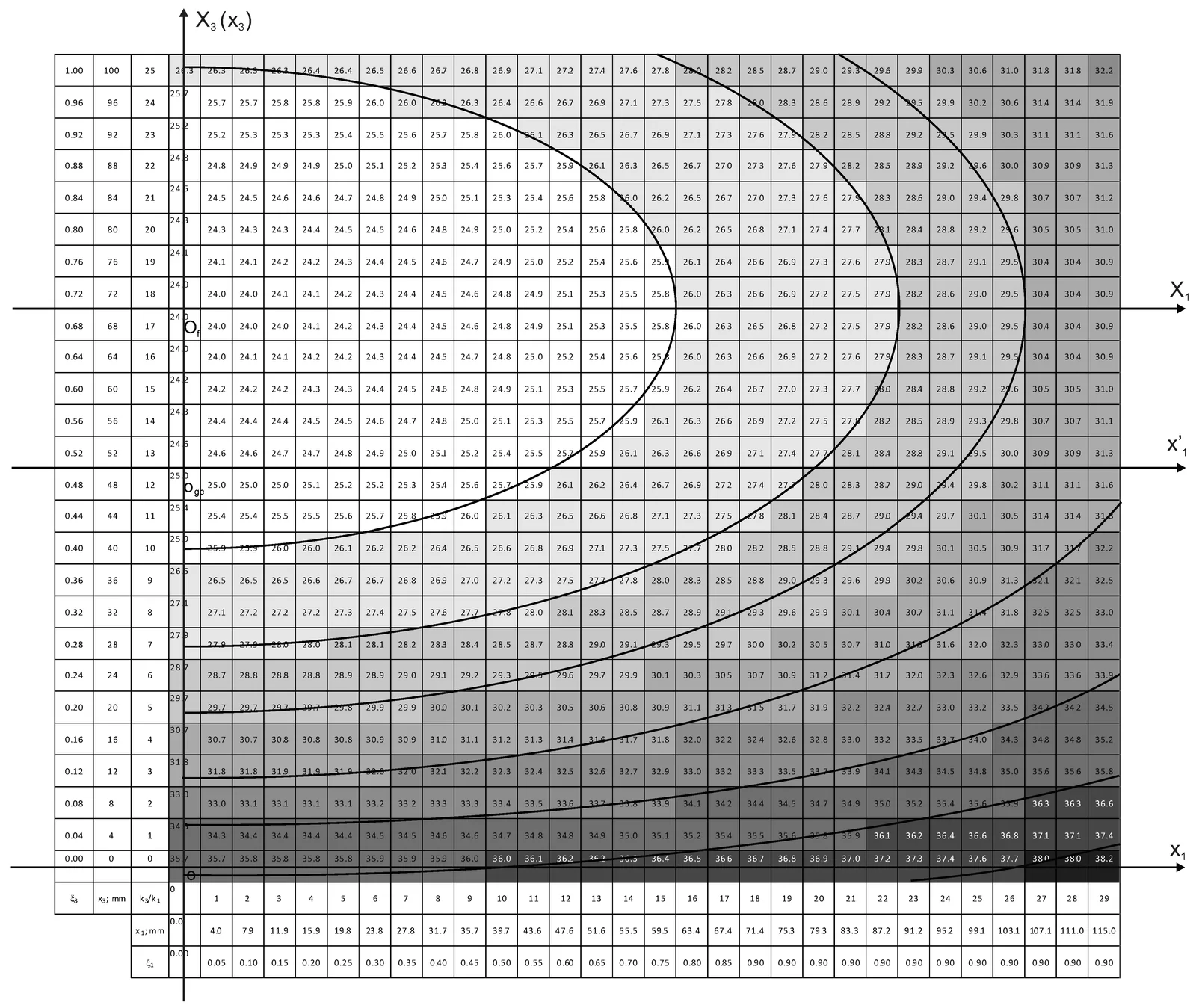
The calculated distribution of elastic modulus in the vertical section X_1_O_f_X_3_ plane; size of elements ΔX_1_ × ΔX_3_ = 4.0 mm × 4.0 mm.

The distribution of the elastic modulus in the vertical section X_1_O_f_X_3_ plane is shown in Figure 5. The modulus is lowest in the central zone, around the foaming center O_f_: E = 24.0 MPa and highest in the bottom right corner of the section: E = 38.2 MPa. The increase in the modulus from the center to the (1) center bottom, (2) center top, (3) bottom right corner, and (4) top right corner is (1) ≈50%, (2) ≈10%, (3) ≈60%, and (4) ≈33%.

The calculated distribution of modulus values, as shown in Figure 4 and Figure 5, is determined by its functional dependency on the density of isotropic foams, as shown in Equation (1). Heat transfer at the contact surfaces with the mold [19] leads to slower gas generation (blowing), premature curing, and higher foam density [1,2,3,4,5], so the material elements located closer to the walls of the relatively cooler mold are denser than those in the center of the block. Compared to other points of the rectangular foam block, heat transfer at the corners is highest due to the three contact surfaces with the mold. A hypothesis is proposed that the density at the bottom corners is higher than at the top corners because less dense material elements rise in the effective media of denser elements due to buoyancy. Furthermore, the bottom of the mold cools faster than the top because the bottom is in contact with the support, while the top is in contact with the surrounding air, which has a comparatively lower thermal conductivity [1,2,3,4,5].

Inside a sealed mold, gas pressure, viscosity of composition, Earth’s gravity, buoyancy, and thermal conductivity are the main factors whose simultaneous action determines the final density/modulus distribution [1,2,3,4,5]. In [19], it was shown that the foaming center O_f_ is located at a height of Δ ≈ 17 mm above the geometric center O_gc_ of an untrimmed (with skins) block. It is of theoretical and practical interest to determine the location of O_f_ in the trimmed block too. The common center O_f_ of the equimodulus lines in the trimmed block is located between the elements with coordinates x_3_ = 68 mm and x_3_ = 72 mm: x_3f_ = 70 ± 4 mm and ξ_3f_ ≈ 0.70 ± 0.04, as shown in Figure 5, which means 0.70 ≈ 0.67 ≈ 2/3 from the bottom of the block and 0.30 ≈ 0.33 ≈ 1/3 from the top. It has yet to be verified whether the ratio 2/3: 1/3 holds for other absolute and relative dimensions of the mold as well.

The expanded uncertainty of density measurements in the range 87 kg/m^3^ ≤ ρ ≤ 114 kg/m^3^ was estimated as U_95%_ ≤ 1.7 kg/m^3^ [19,33,34]. In the given range, the function E = E(ρ) is nearly linear: E = 0.52ρ − 20.8 MPa, as shown in Figure 3, and the corresponding uncertainty of modulus can be estimated as ΔE = 0.52Δρ = 0.52U_95%_ ≤ 0.88 MPa. This estimate does not account for uncertainties associated with regression parameters, model discrepancies, and spatial discretization. Independent verification of the calculated modulus values at selected spatial locations would be necessary by comparing them with experimentally measured values obtained on samples, which are extracted from those same locations.

In 3D space, the equimodulus lines form ellipsoidal equimodulus surfaces. The modulus distribution in all planes, obtained by rotating the X_1_O_f_X_3_ plane about the O_f_X_3_ axis, will be equal to the distribution in the X_1_O_f_X_3_ plane if the volume, enclosed by equidensity surfaces, is an ellipsoid of revolution (spheroid). In this case, x_d_ = x_1_ = x_2_, where x_d_ is the size of an equidensity line in a horizontal section x_1_ox_2_ along the diagonal axis ox_d_ of the block [19] and x_1_, x_2_ is the size along axes ox_1_ and ox_2_. Let us assume that the relative difference Q = (x_d_ − x_1_)/x_1_ ≤ 15–20% is acceptable for practical purposes, such as preparing test samples. The Q value characterizes the degree of deviation of the equidensity line from a circle due to the influence of the rectangular cross-section of the mold. An analysis of density maps given in [19] showed that the largest value of x_1_, for which x_d_ ≈ x_1_ in all six horizontal layers, in terms of Q ≤ 15–20%, is x_1_ ≈ 100 mm. The volume enclosed by the equidensity surfaces can still be considered as a spheroid for all x_1_ ≤ 100 mm (ξ_1_ ≤ 0.87). The equidensity surfaces will be exact spheroids for PU foams produced in a sealed mold with a circular cross-section (a cylinder).

The distribution maps in Figure 4 and Figure 5 include several such partition elements, whose shading contradicts the numerical value, specified in the frame of the element. For example, for the element where x_1_ = 63.3 mm and x_3_ = 66.7 mm in Figure 5, the modulus value is 26.0 MPa, while the shading corresponds to modulus zone № 1: 24.0 MPa ≤ E < 26 MPa, as shown in Table 2. The value assigned to the element is E = 25.993 MPa < 26 MPa, which corresponds to zone № 1, but is rounded to 26.0 MPa to fit within the limited element frame.

When using rigid PU foams with density and modulus gradients in engineering products such as sandwich panel cores, molded parts, insulation blocks, etc., it is essential to account for the changes in density and modulus that can adversely affect the dimensional stability of products and contribute to local stress concentrations, failure initiation sites, crack formation, etc. These factors must be considered during mold design, the selection of processing parameters, and the planning of samples’ extraction for mechanical testing.

### 3.3. Elastic Modulus in the Central Vertical Section

**Figure 6:**
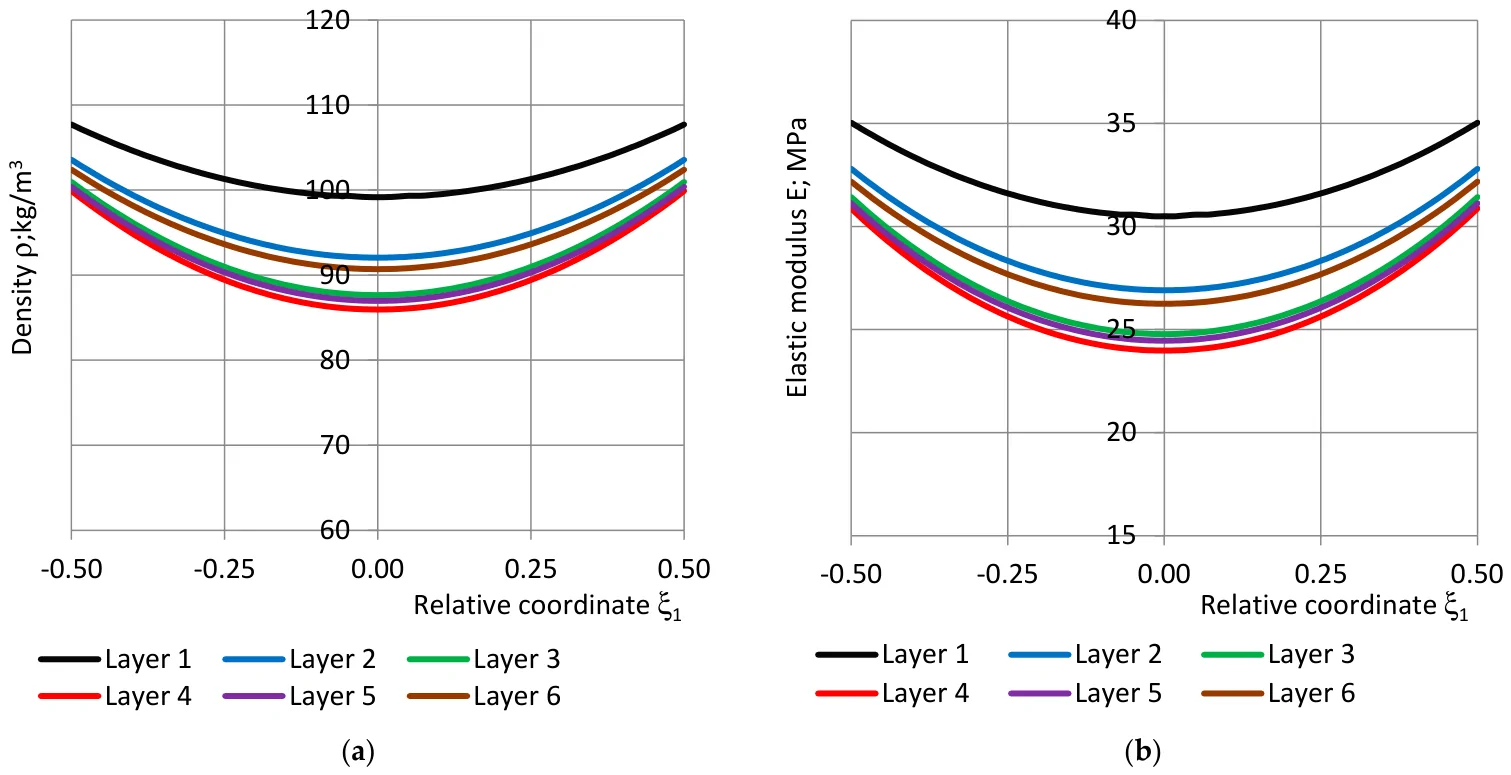
Density (**a**) and elastic modulus (**b**) in the section ABCD of the block.

The grading of density and elastic modulus in the central vertical section ABCD (x_1_ox_3_) of the block, as shown in Figure 1, is shown in Figure 6 depending on relative coordinate ξ_1_ = x_1_/L_1_ = x_1_ mm/230 mm for discrete values of x_3_, which correspond to the height middle of the six horizontal layers, as shown in Table 4 [19]. The values of density and modulus correspond to row № 0 of the density and modulus maps of the horizontal layers [19] and Figures S2–S4 in the Supplementary Materials.

It can be seen that the density and elastic modulus are increasing in the direction from the center ξ_1_ = 0.0 to the sides ξ_1_ = ±0.50 of the section ABCD for all layers № 1–6. The relative increase in density is in the range of 9% ≤ R_ρ_ ≤ 16%, and in modulus it is in the range of −15% ≤ R_E_ ≤ 29%, as shown in Table 4. The greatest increase is observed in layer № 4 (red curves), which includes the foaming center O_f_, and the smallest increase is observed in the bottom layer № 1 (black curves).

### 3.4. Aspect Ratios of the Equidensity and Equimodulus Ellipses

The semi-axes “a” and “b” of equidensity ellipses № 1–9, determined from the density map of the X_1_O_f_X_3_ section at size of elements 4 mm × 4 mm, are given in Table 5.

**Figure 7:**
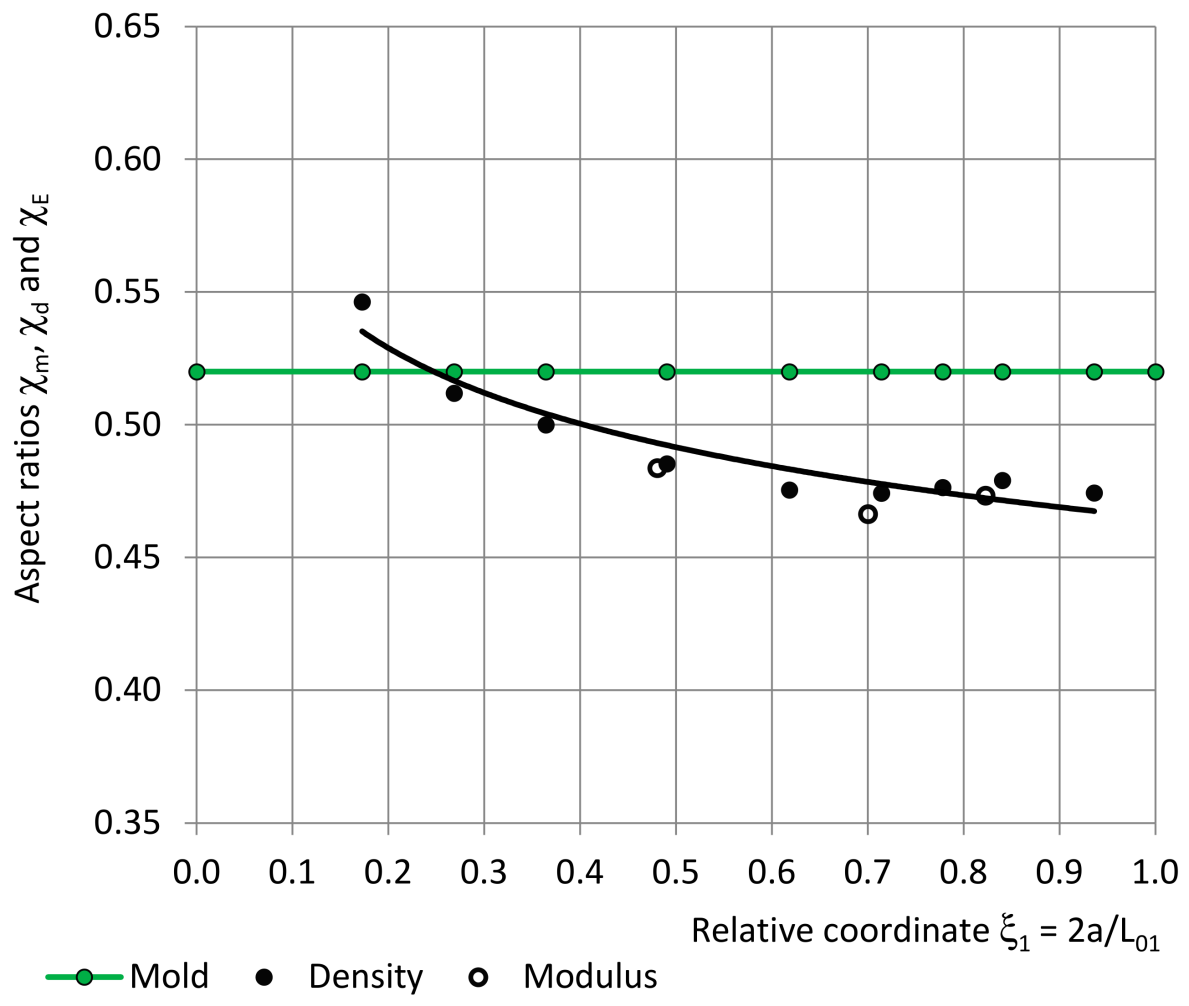
Dependence of the aspect ratios χ_m_, χ_d_ and χ_E_ of the (1) mold (•), (2) equidensity ellipses (•) and (3) equimodulus ellipses (ο) on the relative coordinate ξ_1_ = X_1_/(L_01_/2).

The dependence of the aspect ratio χ_d_ = b/a on the relative coordinate χ_d_ = χ_d_(ξ_1_) is presented in Figure 7, along with the aspect ratios of the mold and equimodulus ellipses.

The data of χ_d_ are approximated by a power function of the coordinate X_1_:

(5) $$\chi_{d}=C{\left(\xi_{1}\right)}^{-0.08}=C{\left[\frac{X_{1}}{L_{01}/2}\right]}^{-0.08},\;\mathrm{where}\;C\;=\;0.465.$$

Since the aspect ratio of the mold is constant at χ_m_ = L_03_/L_01_ = 0.52 for all ξ_1_ = 2X_1_/L_01_, the constant C can be expressed as C = C_1_χ_m_, where C_1_ = 0.894:

(6) $$\chi_{d}={0.894\chi_{m}\left(\frac{L_{01}}{2X_{1}}\right)}^{0.08}={0.894\frac{L_{03}}{L_{01}}\left(\frac{L_{01}}{2X_{1}}\right)}^{0.08}.$$

The empiric Equation (6) describes the dependence of χ_d_ on X_1_ for a given aspect ratio of the mold χ_m_ = 0.52. It can be concluded that with an increase in the relative coordinate ξ_1_ the aspect ratio χ_d_ decreases non-linearly. For most of the dimensionless coordinate ξ_1_ > 0.25, the aspect ratio of the equidensity ellipses is less than that of the mold: χ_d_ < χ_m_. In the limiting case of peripheral ellipses, when X_1_ tends to L_01_/2, the aspect ratio χ_d_ tends to 0.465. In the case of central ellipses, when the coordinate X_1_ becomes comparable to the size of partition element ΔX_1_ = 4 mm (Δξ_1_ = 3%), then χ_d_ = 0.62. As the eccentricity e increases, the ellipses become flatter; the closer to e = 1.0 it gets, the flatter the ellipses. It should be noted that Equation (6) describes the dependence of χ_d_ on X_1_ for the given aspect ratio of the rectangular mold χ_m_ = 0.52, as well as for the given chemical composition and production technology. Under different foaming conditions, the form of equation and the numerical values of constants may differ from those presented here.

Theoretically, the aspect ratio of the equimodulus lines is equal to that of the equidensity lines, χ_E_ = χ_d_, but the finite dimensions of the partition elements introduce uncertainty in determining the aspect ratio from the equilines’ maps and χ_E_ ≈ χ_d_, as shown in Figure 7. To estimate the shape and size of the equidensity and equimodulus lines in the very center of the block (ξ_1_ < 15–20%), density data measured on smaller samples than those used in [19] are needed, as well as distribution maps with even smaller elements.

### 3.5. Mathematical Model of the Equimodulus Lines at Any Point of the Vertical Section

**Figure 8:**
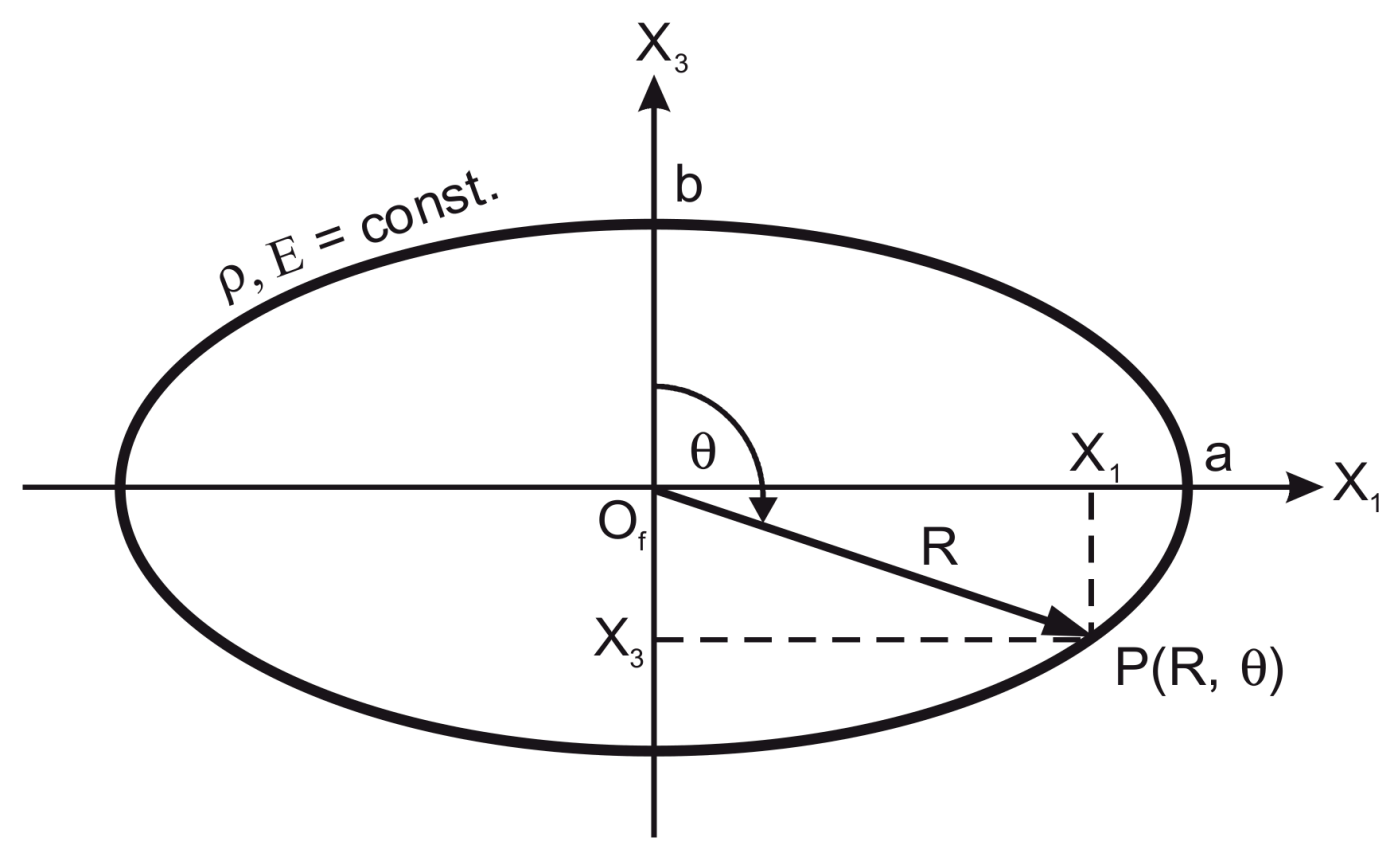
Point P(R, θ) in the X_1_O_f_X_3_ plane.

So far, the equidensity and equimodulus lines have been plotted for finite-size partition elements. Equation (6) allows us to model density and modulus of elasticity at any point of the vertical section X_1_O_f_X_3_ of the PU foams’ block. For this purpose, an arbitrary point P(R, θ) in the X_1_O_f_X_3_ plane is considered, where R, θ are the spherical coordinates, as shown in Figure 8.

The point P(R, θ) belongs to a certain equidensity ellipse, which is defined by the equation

(7) $$\frac{X_{1}^{2}}{a^{2}}+\frac{X_{3}^{2}}{b^{2}}=1.0,\mathrm{where}\;X_{1}=\mathrm{Rsin\theta}\;\mathrm{and}\;X_{3}=-\mathrm{Rcos\theta}.$$

The aspect ratio of the ellipse equals χ_d_ = b/a; then, semi-major axis a can be expressed as:

(8) $$a(R,\theta )=\frac{R}{\chi_{d}}\sqrt{\chi_{d}^{2}\sin^{2}\theta +\cos^{2}\theta },$$

Substituting Equation (6) into Equation (8) leads to

(9) $$a(R,\theta )=\frac{RL_{01}}{{C_{1}\frac{L_{03}}{L_{01}}\left(\frac{L_{01}}{2\mathrm{Rsin}\theta }\right)}^{0.08}}\sqrt{{\left[{C_{1}\frac{L_{03}}{L_{01}}\left(\frac{L_{01}}{2\mathrm{Rsin}\theta }\right)}^{0.08}\right]}^{2}\sin^{2}\theta +\cos^{2}\theta },$$

where R, θ, C_1_, L_01_, and L_03_ are known. The expression (9) and relationship b = aχ_d_ permit determining of the semi-axes “a” and “b” of the equidensity ellipse for any point P(R, θ) of the cross-section of the block.

The density in points (X_1_, X_3_ = 0 mm) on O_f_X_1_ axis corresponds to row № 0 of the expanded density matrix of horizontal layer № 4 given in Table S15 of the Supplementary Materials [19]. The experimental density data in row № 0 (i.e., along OX_1_ axis) of layer № 4 were approximated by a second-order polynomial

ρ(ξ_1_) = 13.96(ξ′_1_)^2^ − 0.014ξ′_1_ + 85.96; (10)

where ξ′_1_ = X_1_/(L_1_/2) = 2X_1_/(L_01_ − 2ΔL) and ΔL = 10 mm is the trimming allowance. Then, the density along OX_1_ axis can be expressed as:

(11) $$\rho (X_{1},\;X_{3}\;=\;0)\;=\;55.84\;{\left[\frac{X_{1}}{L_{01}-2∆L}\right]}^{2}-\;0.028\frac{X_{1}}{L_{01}-2∆L}+85.96.$$

The density along any equidensity ellipse with the semi-major axis X_1_ = a is calculated as

(12) $$\rho (R,\;\theta )=55.84\;{\left[\frac{a(R,\theta )}{L_{01}-2∆L}\right]}^{2}-0.028\frac{a(R,\theta )}{L_{01}-2∆L}+85.96,$$

where a(R, θ) is determined from Equation (9). Since density is constant along an equidensity line, Equation (1) permits calculating the corresponding modulus value:

(13) $$E(R,\;\theta )\;=\;A\rho (R,\;\theta {)}^{d}\;=\;A{\left\{55.84{\left[\frac{a(R,\theta )}{L_{01}-2∆L}\right]}^{2}-0.028\frac{a(R,\theta )}{L_{01}-2∆L}+85.96\right\}}^{d},$$

where A = 0.0135 and d = 1.680. Since the foams are locally isotropic with **a** Poisson’s ratio ν ≈ 0.33, as shown in Table 3, the shear and bulk moduli [39] at any point of the vertical section X_1_OX_3_ can be expressed as

(14) $$G\left(R,\theta \right)=\frac{E\left(R,\theta \right)}{2\left(1+\nu \right)},\;K(R,\theta )=\frac{E(R,\theta )}{3(1-2\nu )}.$$

It is concluded that the moduli E, G and K at a given point in the vertical section X_1_O_f_X_3_ of a rigid PU foam block, produced in a sealed rectangular mold, at overpressure, are determined by the dimensions of the mold, the parameters A and d of the given foam composition, and the density distribution in row № 0 of the horizontal layer, which includes the foaming center O_f_. The derived equations for calculation of the moduli E, G, and K are valid for rigid PU foams produced at the investigated conditions, under the assumption of local isotropy.

## 4. Conclusions

A method was developed for evaluating the distribution of the elastic modulus using the density distribution maps for a rigid PU foam block, which is produced in a sealed mold, at overpressure. The empirical relationship between the elastic modulus and density of the PU foam composition was studied experimentally in compression, and the constants of the model function were determined. This made it possible to calculate the values of the modulus and plot the modulus distribution maps for vertical and horizontal cross-sections of the PU foam block, produced via confined foaming in a sealed mold. The modulus distribution was found to be circular in horizontal cross-sections and elliptical in vertical cross-sections of the block. It can be concluded that the elliptical symmetry of the density distribution leads to symmetry of the same kind in the elastic properties of rigid PU foams. The experimentally obtained Poisson’s ratio values supported the conclusion about local isotropy for the produced PU foams.

The functional relationship between the aspect ratio of the mold and of the equidensity and equimodulus ellipses was assessed. It was found that for most of the block, the aspect ratio of the equidensity ellipses is smaller than that of the mold. The assessment of the trends is limited by the accuracy of the experimental data of density and the finite size of the partition elements in the distribution maps.

The proposed methods and acquired results apply to the investigated foam formulation, mold geometry, and processing conditions. The modulus distribution maps are model-based predictions which require an independent experimental spatial validation before broader generalization. Further studies should focus on the independent verification of the calculated modulus values, as well as on the dependence of effective elastic moduli on the size/location of the testing samples and the loading direction.

## Figures and Tables

**Table 1:** Chemical composition of the rigid PU foams.

Number	Component	Trade Name	Producer	pbw
1	Polyether polyol	Lupranol 3000	BASF SE (Ludwigshafen, Germany)	45
2	Polyester polyol	Lupraphen 1901/1		30
3	Chain extender	Diethylene glycol	Sigma-Aldrich Co. LLC (St. Louis, MO, USA)	25
4	Flame retardant	Tris(1-chloro-2-propyl) phosphate	Albemarle Corp. (Charlotte, NC, USA)	15
5	Surfactant	Silicone L6915LV	Momentive Performance Materials (Niskayuna, NY, USA)	1.5
6	Catalyst package	PC CAT NP10	Air Products and Chemicals Inc. (Allentown, PA, USA)	0.3
7		Kosmos 19	Evonik Industries AG (Essen, Germany)	
8	Physical blowing agent	Pentane	Sigma-Aldrich Co. LLC (St. Louis, MO, USA)	25
9	Chemical blowing agent	Distilled water		0.1
10	Polyisocyanate	Desmodur 44V20L	Covestro AG (Leverkusen, Germany)	168

**Table 2:** The shade legend of equimodulus zones.

Number of a Zone	1	2	3	4	5	6	7	8
Modulus range; MPa	24–26	26–28	28–30	30–32	32–34	34–36	36–38	38–40
Shade								

**Table 3:** The results of PU foams’ compression tests.

N	Density ρ; kg/m^3^	Modulus E; MPa	Poisson’s Ratio ν_31_	Average Poisson’s Ratio ν_31av_
1	45.2 ± 0.9 (2%)	8.3 ± 0.7 (8%)	0.36 ± 0.04 (11%)	0.35 ± 0.01
2	49.3 ± 1.5 (3%)	9.0 ± 0.4 (5%)	0.34 ± 0.02 (6%)	
3	57.5 ± 0.6 (1%)	12.6 ± 0.9 (7%)	0.35 ± 0.02 (6%)	
4	89.1 ± 0.9 (1%)	24.6 ± 1.4 (5%)	0.33 ± 0.01 (3%)	0.33 ± 0.01
5	91.3 ± 1.8 (2%)	26.9 ± 1.0 (4%)	0.32 ± 0.03 (9%)	
6	97.0 ± 1.9 (2%)	30.9 ± 1.7 (6%)	0.33 ± 0.03 (9%)	

**Table 4:** Characteristics of the grading of density and elastic modulus in the vertical section ABCD of the block.

Number of a Horizontal Layer	Coordinate x_3_; mm	Relative Coordinate ξ_3_	Increase in Density Δρ; kg/m^3^	Relative Increase in Density R_ρ_; %	Increase in Modulus ΔE; MPa	Relative Increase in Modulus R_E_; %
6	91.9	0.92	11.7	13	5.9	23
5	75.2	0.75	13.4	15	6.7	27
4	58.5	0.58	14.0	16	6.9	29
3	41.8	0.42	13.3	15	6.6	27
2	25.1	0.25	11.5	13	5.9	22
1	8.4	0.08	8.5	9	4.5	15

**Table 5:** Parameters of the equidensity ellipses.

N	Density ρ; kg/m^3^	Semi-Axes; mm		Aspect Ratio χ_d_ = b/a	Linear Eccentricity c; mm	Eccentricity e	Relative Coordinate ξ_1_
		a	b				
1	86.5	21.6	11.8	0.55	18.1	0.84	0.17
2	87.1	33.5	17.2	0.51	28.8	0.86	0.27
3	88.2	45.5	22.8	0.50	39.4	0.87	0.36
4	90.0	61.3	29.7	0.49	53.6	0.87	0.49
5	92.3	77.3	36.7	0.48	68.0	0.88	0.62
6	94.3	89.2	42.3	0.47	78.6	0.88	0.71
7	95.9	97.2	46.3	0.48	85.5	0.88	0.78
8	98.1	105.0	50.3	0.48	92.2	0.88	0.84
9	101.0	117.0	55.5	0.47	103.0	0.88	0.94

## Data Availability

Data is contained within the Supplementary Materials to the article.

## Supplementary Materials

The following supporting information can be downloaded at https://www.mdpi.com/article/doi/s1, Figure S1: The map of density distribution in the vertical section X_1_O_f_X_3_, where X_1_O_f_X_3_ and x′_1_o_gc_x′_3_ are local coordinate systems with origin in the (a) foaming centre O_f_ and (b) geometric centre o_gc_ of the block; size of elements ΔX_1_ × ΔX_3_ = 4 mm × 4 mm; Figure S2: The calculated maps of elasticity modulus’ distribution in horizontal layers (a) № 1 and (b) № 2 (in the plane parallel to X_1_O_f_X_2_ plane; size of elements ΔX_1_ × ΔX_2_ = 5.6 mm × 5.6 mm); Figure S3: The calculated maps of elasticity modulus’ distribution in horizontal layers (a) № 3 and (b) № 4 (in the plane parallel to X_1_O_f_X_2_ plane; size of elements ΔX_1_ × ΔX_2_ = 5.6 mm × 5.6 mm); Figure S4: The calculated maps of elasticity modulus’ distribution in horizontal layers (a) № 5 and (b) № 6 (in the plane parallel to X_1_O_f_X_2_ plane; size of elements ΔX_1_ × ΔX_2_ = 5.6 mm × 5.6 mm).
