# Supplementary material for: Elastic Modulus Distribution in Rigid PU Foams of Elliptically Graded Density
Source: Polymers (Basel). 2026 Oct 16;18(20):2549. doi: 10.3390/polym18202549 (PMC13709305; doi:10.3390/polym18202549)
Supplement: Supplementary file 1 [file polymers-18-02549-s001.zip › polymers-4605054-supplementary.pdf]

polymers

MDPI

Supplementary Materials

Elastic Modulus Distribution in Rigid PU Foams of Elliptically Graded Density

Ilze Beverte and Ugis Cabulis *

Latvian State Institute of Wood Chemistry, Dzērbenes Street 27, LV-1006 Riga, Latvia; a09660418@gmail.com
* Correspondence: ugis.cabulis@kki.lv; Tel.: +371-29-27-26-58

 https://doi.org/10.3390/polym18202549

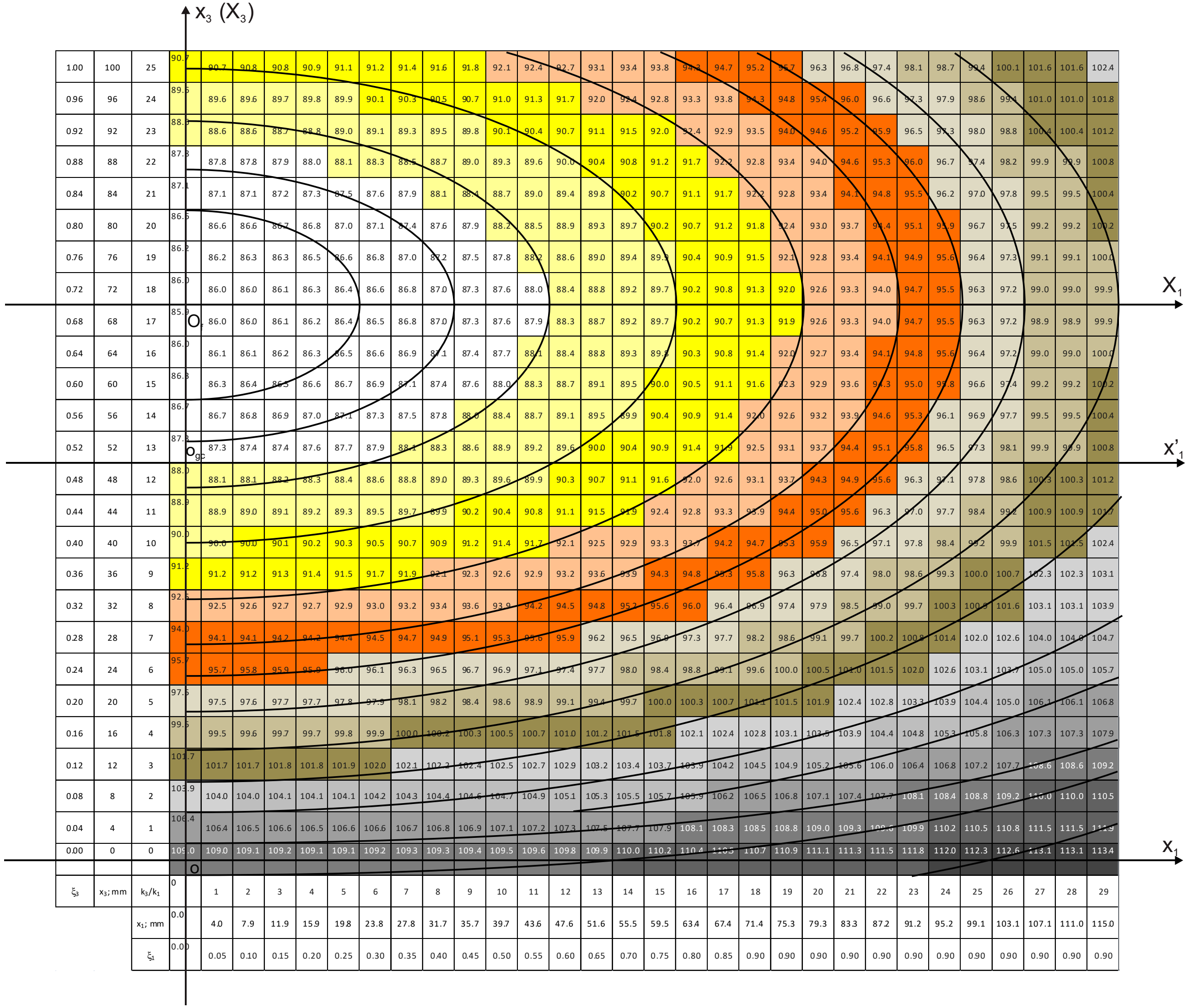

Figure S1. The map of density distribution in the vertical section $X_1O_fX_3$, where $X_1O_fX_3$ and $x'_1o_{gc}x'_3$ are local coordinate systems with origin in the a) foaming centre O_f and b) geometric centre o_{gc} of the block; size of elements $\Delta X_1 \times \Delta X_3 = 4$ mm $\times$ 4 mm.

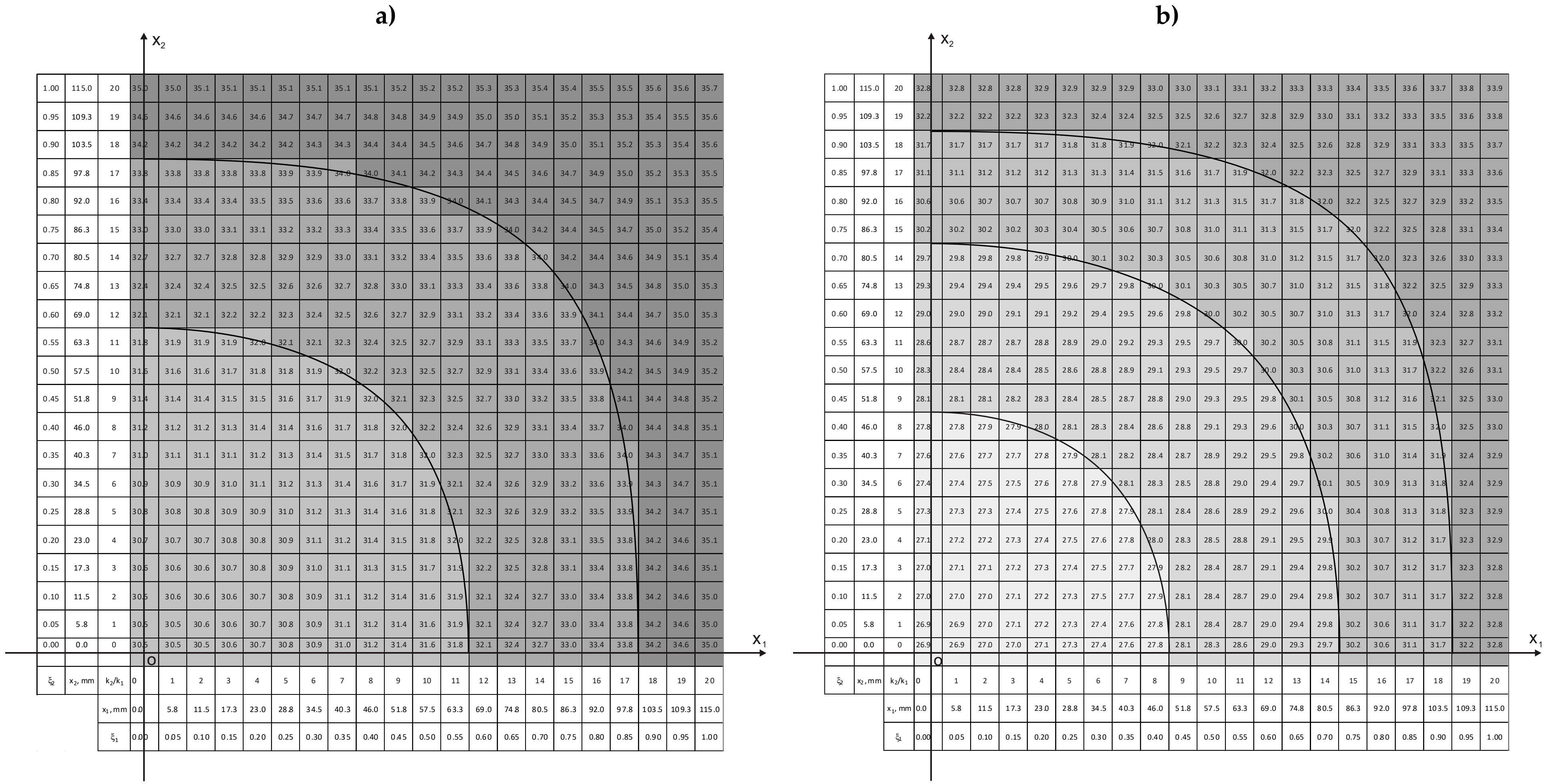

Figure S2. The calculated maps of elasticity modulus' distribution in horizontal layers **a)** № 1 and **b)** № 2 (in the plane parallel to $X_1O_fX_2$ plane; size of elements $\Delta X_1 \times \Delta X_2$ = 5.6 mm × 5.6 mm).

a)

ξ_2	x_2, mm	k_2/k_1	0	1	2	3	4	5	6	7	8	9	10	11	12	13	14	15	16	17	18	19	20
1.00	115.0	20	31.4	31.4	31.4	31.5	31.5	31.5	31.5	31.6	31.6	31.7	31.7	31.8	31.9	31.9	32.0	32.1	32.2	32.3	32.4	32.5	32.6
0.95	109.3	19	30.8	30.8	30.8	30.8	30.8	30.9	30.9	31.0	31.0	31.1	31.2	31.3	31.4	31.5	31.6	31.7	31.9	32.0	32.2	32.3	32.5
0.90	103.5	18	30.1	30.1	30.1	30.2	30.2	30.3	30.3	30.4	30.5	30.6	30.7	30.8	30.9	31.1	31.2	31.4	31.6	31.8	32.0	32.2	32.4
0.85	97.8	17	29.5	29.5	29.6	29.6	29.6	29.7	29.8	29.9	30.0	30.1	30.2	30.4	30.5	30.7	30.9	31.1	31.3	31.5	31.8	32.0	32.3
0.80	92.0	16	29.0	29.0	29.0	29.0	29.1	29.2	29.3	29.4	29.5	29.6	29.8	29.9	30.1	30.3	30.5	30.8	31.0	31.3	31.6	31.9	32.2
0.75	86.3	15	28.4	28.5	28.5	28.5	28.6	28.7	28.8	28.9	29.0	29.2	29.3	29.5	29.7	30.0	30.2	30.5	30.8	31.1	31.4	31.7	32.1
0.70	80.5	14	28.0	28.0	28.0	28.1	28.1	28.2	28.3	28.4	28.6	28.8	29.0	29.2	29.4	29.6	29.9	30.2	30.5	30.9	31.2	31.6	32.0
0.65	74.8	13	27.5	27.5	27.6	27.6	27.7	27.8	27.9	28.0	28.2	28.4	28.6	28.8	29.1	29.4	29.6	30.0	30.3	30.7	31.1	31.5	31.9
0.60	69.0	12	27.1	27.1	27.1	27.2	27.3	27.4	27.5	27.7	27.8	28.0	28.3	28.5	28.8	29.1	29.4	29.7	30.1	30.5	30.9	31.4	31.9
0.55	63.3	11	26.7	26.7	26.8	26.8	26.9	27.0	27.2	27.3	27.5	27.7	28.0	28.2	28.5	28.8	29.2	29.5	29.9	30.4	30.8	31.3	31.8
0.50	57.5	10	26.4	26.4	26.4	26.5	26.6	26.7	26.9	27.0	27.2	27.4	27.7	28.0	28.3	28.6	29.0	29.3	29.8	30.2	30.7	31.2	31.7
0.45	51.8	9	26.1	26.1	26.1	26.2	26.3	26.4	26.6	26.7	26.9	27.2	27.4	27.7	28.0	28.4	28.8	29.2	29.6	30.1	30.6	31.1	31.7
0.40	46.0	8	25.8	25.8	25.9	25.9	26.0	26.2	26.3	26.5	26.7	26.9	27.2	27.5	27.8	28.2	28.6	29.0	29.5	30.0	30.5	31.0	31.6
0.35	40.3	7	25.6	25.6	25.6	25.7	25.8	25.9	26.1	26.3	26.5	26.7	27.0	27.3	27.7	28.0	28.4	28.9	29.4	29.9	30.4	31.0	31.6
0.30	34.5	6	25.4	25.4	25.4	25.5	25.6	25.7	25.9	26.1	26.3	26.6	26.9	27.2	27.5	27.9	28.3	28.8	29.3	29.8	30.3	30.9	31.5
0.25	28.8	5	25.2	25.2	25.2	25.3	25.4	25.6	25.7	25.9	26.2	26.4	26.7	27.0	27.4	27.8	28.2	28.7	29.2	29.7	30.3	30.9	31.5
0.20	23.0	4	25.0	25.1	25.1	25.2	25.3	25.4	25.6	25.8	26.0	26.3	26.6	26.9	27.3	27.7	28.1	28.6	29.1	29.6	30.2	30.8	31.5
0.15	17.3	3	24.9	24.9	25.0	25.1	25.2	25.3	25.5	25.7	25.9	26.2	26.5	26.8	27.2	27.6	28.1	28.5	29.0	29.6	30.2	30.8	31.5
0.10	11.5	2	24.8	24.9	24.9	25.0	25.1	25.2	25.4	25.6	25.9	26.1	26.4	26.8	27.1	27.6	28.0	28.5	29.0	29.6	30.1	30.8	31.4
0.05	5.8	1	24.8	24.8	24.9	24.9	25.1	25.2	25.4	25.6	25.8	26.1	26.4	26.7	27.1	27.5	28.0	28.5	29.0	29.5	30.1	30.8	31.4
0.00	0.0	0	24.8	24.8	24.8	24.9	25.0	25.2	25.4	25.6	25.8	26.1	26.4	26.7	27.1	27.5	28.0	28.4	29.0	29.5	30.1	30.8	31.4
		x_1, mm	0.0	5.8	11.5	17.3	23.0	28.8	34.5	40.3	46.0	51.8	57.5	63.3	69.0	74.8	80.5	86.3	92.0	97.8	103.5	109.3	115.0
		ξ_1	0.00	0.05	0.10	0.15	0.20	0.25	0.30	0.35	0.40	0.45	0.50	0.55	0.60	0.65	0.70	0.75	0.80	0.85	0.90	0.95	1.00

b)

ξ_2	x_2, mm	k_2/k_1	0	1	2	3	4	5	6	7	8	9	10	11	12	13	14	15	16	17	18	19	20
1.00	115.0	20	30.9	30.9	30.9	30.9	30.9	31.0	31.0	31.0	31.1	31.1	31.2	31.2	31.3	31.4	31.4	31.5	31.6	31.7	31.8	31.9	32.0
0.95	109.3	19	30.2	30.2	30.2	30.2	30.2	30.3	30.3	30.4	30.4	30.5	30.6	30.7	30.8	30.9	31.0	31.1	31.3	31.4	31.6	31.7	31.9
0.90	103.5	18	29.5	29.5	29.5	29.6	29.6	29.7	29.7	29.8	29.9	30.0	30.1	30.2	30.3	30.5	30.6	30.8	31.0	31.1	31.3	31.6	31.8
0.85	97.8	17	28.9	28.9	28.9	29.0	29.0	29.1	29.1	29.2	29.3	29.5	29.6	29.7	29.9	30.1	30.3	30.5	30.7	30.9	31.1	31.4	31.7
0.80	92.0	16	28.3	28.3	28.3	28.4	28.4	28.5	28.6	28.7	28.8	29.0	29.1	29.3	29.5	29.7	29.9	30.1	30.4	30.7	31.0	31.3	31.6
0.75	86.3	15	27.8	27.8	27.8	27.9	27.9	28.0	28.1	28.2	28.4	28.5	28.7	28.9	29.1	29.3	29.6	29.9	30.1	30.5	30.8	31.1	31.5
0.70	80.5	14	27.3	27.3	27.3	27.4	27.4	27.5	27.6	27.8	27.9	28.1	28.3	28.5	28.7	29.0	29.3	29.6	29.9	30.3	30.6	31.0	31.4
0.65	74.8	13	26.8	26.8	26.9	26.9	27.0	27.1	27.2	27.4	27.5	27.7	27.9	28.2	28.4	28.7	29.0	29.3	29.7	30.1	30.5	30.9	31.4
0.60	69.0	12	26.4	26.4	26.4	26.5	26.6	26.7	26.8	27.0	27.1	27.3	27.6	27.8	28.1	28.4	28.7	29.1	29.5	29.9	30.3	30.8	31.3
0.55	63.3	11	26.0	26.0	26.0	26.1	26.2	26.3	26.5	26.6	26.8	27.0	27.3	27.5	27.8	28.2	28.5	28.9	29.3	29.7	30.2	30.7	31.2
0.50	57.5	10	25.6	25.7	25.7	25.8	25.9	26.0	26.1	26.3	26.5	26.7	27.0	27.3	27.6	27.9	28.3	28.7	29.1	29.6	30.1	30.6	31.2
0.45	51.8	9	25.3	25.3	25.4	25.4	25.5	25.7	25.8	26.0	26.2	26.5	26.7	27.0	27.3	27.7	28.1	28.5	29.0	29.5	30.0	30.5	31.1
0.40	46.0	8	25.0	25.1	25.1	25.2	25.3	25.4	25.6	25.7	26.0	26.2	26.5	26.8	27.1	27.5	27.9	28.4	28.8	29.3	29.9	30.4	31.1
0.35	40.3	7	24.8	24.8	24.9	24.9	25.0	25.2	25.3	25.5	25.7	26.0	26.3	26.6	27.0	27.4	27.8	28.2	28.7	29.2	29.8	30.4	31.0
0.30	34.5	6	24.6	24.6	24.6	24.7	24.8	25.0	25.1	25.3	25.6	25.8	26.1	26.5	26.8	27.2	27.6	28.1	28.6	29.1	29.7	30.3	31.0
0.25	28.8	5	24.4	24.4	24.5	24.5	24.6	24.8	25.0	25.2	25.4	25.7	26.0	26.3	26.7	27.1	27.5	28.0	28.5	29.1	29.7	30.3	31.0
0.20	23.0	4	24.2	24.3	24.3	24.4	24.5	24.6	24.8	25.0	25.3	25.5	25.9	26.2	26.6	27.0	27.4	27.9	28.4	29.0	29.6	30.2	30.9
0.15	17.3	3	24.1	24.1	24.2	24.3	24.4	24.5	24.7	24.9	25.2	25.4	25.8	26.1	26.5	26.9	27.4	27.9	28.4	29.0	29.6	30.2	30.9
0.10	11.5	2	24.1	24.1	24.1	24.2	24.3	24.5	24.6	24.9	25.1	25.4	25.7	26.0	26.4	26.9	27.3	27.8	28.3	28.9	29.5	30.2	30.9
0.05	5.8	1	24.0	24.0	24.1	24.1	24.3	24.4	24.6	24.8	25.1	25.3	25.7	26.0	26.4	26.8	27.3	27.8	28.3	28.9	29.5	30.2	30.9
0.00	0.0	0	24.0	24.0	24.0	24.1	24.2	24.4	24.6	24.8	25.0	25.3	25.6	26.0	26.4	26.8	27.3	27.8	28.3	28.9	29.5	30.2	30.9
		x_1, mm	0.0	5.8	11.5	17.3	23.0	28.8	34.5	40.3	46.0	51.8	57.5	63.3	69.0	74.8	80.5	86.3	92.0	97.8	103.5	109.3	115.0
		ξ_1	0.00	0.05	0.10	0.15	0.20	0.25	0.30	0.35	0.40	0.45	0.50	0.55	0.60	0.65	0.70	0.75	0.80	0.85	0.90	0.95	1.00

Figure S3. The calculated maps of elasticity modulus' distribution in horizontal layers **a)** № 3 and **b)** № 4 (in the plane parallel to $X_1O_fX_2$ plane; size of elements $\Delta X_1 \times \Delta X_2$ = 5.6 mm × 5.6 mm).

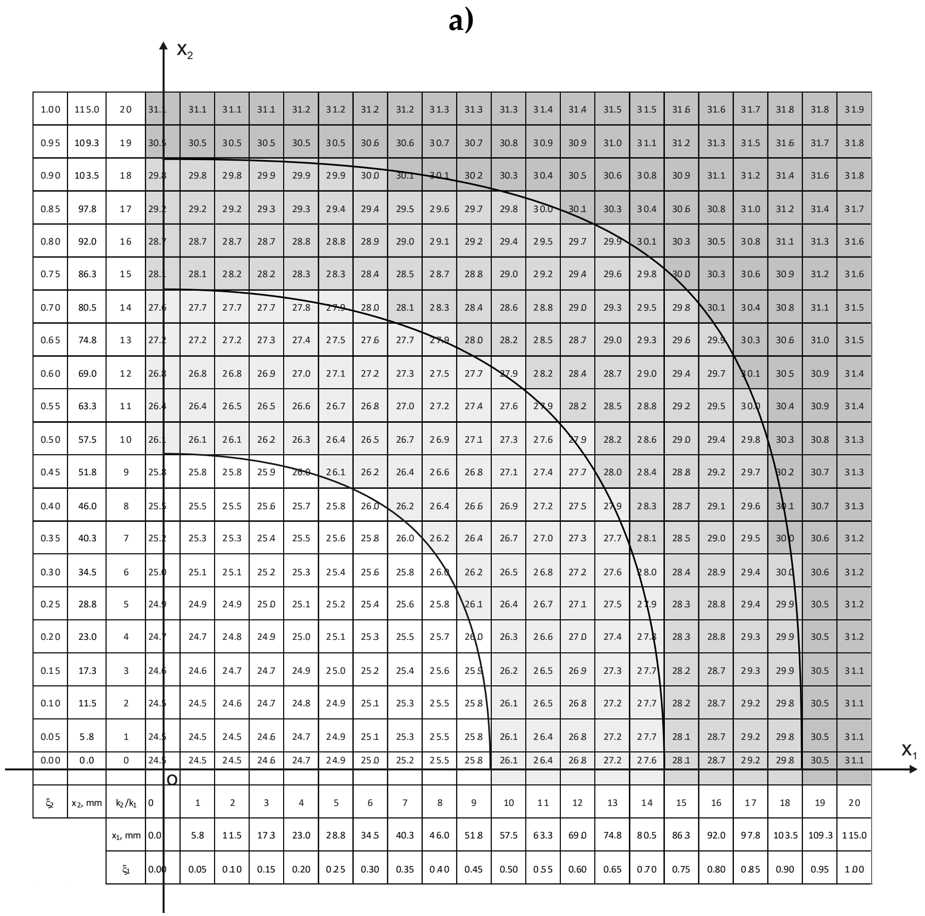

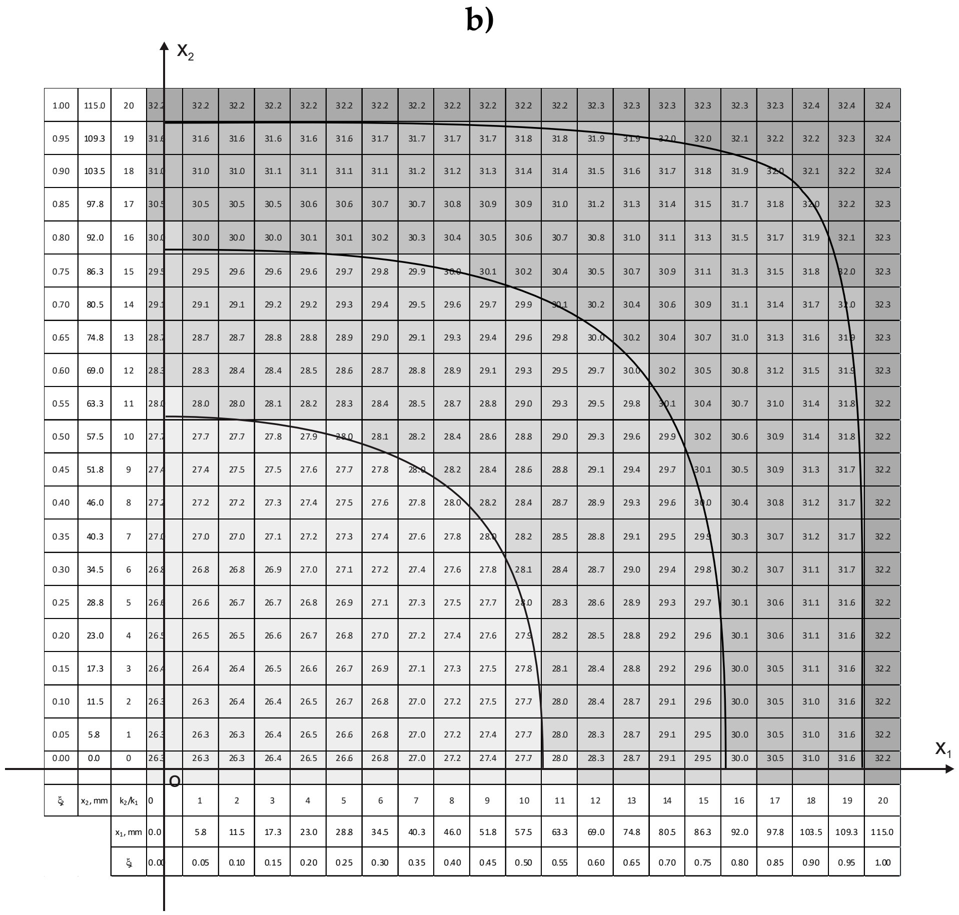

Figure S4. The calculated maps of elasticity modulus' distribution in horizontal layers **a)** № 5 and **b)** № 6 (in the plane parallel to $X_1O_fX_2$ plane; size of elements $\Delta X_1 \times \Delta X_2$ = 5.6 mm × 5.6 mm).
